# *Rhodiola/Cordyceps*-Based Herbal Supplement Promotes Endurance Training-Improved Body Composition But Not Oxidative Stress and Metabolic Biomarkers: A Preliminary Randomized Controlled Study

**DOI:** 10.3390/nu11102357

**Published:** 2019-10-03

**Authors:** Yi-Hung Liao, Yi-Chen Chao, Brenton Yim-Quan Sim, Hui-Mei Lin, Mu-Tsung Chen, Chung-Yu Chen

**Affiliations:** 1 Department of Exercise and Health Science, National Taipei University of Nursing and Health Sciences, Taipei City 112, Taiwan; 2 Department of Exercise and Health Sciences, University of Taipei, Taipei City 111, Taiwan; 3 Department of Aquatic Sports, University of Taipei, Taipei City 111, Taiwan; 4 School of Liberal Education, Shih Chien University, Taipei City 104, Taiwan

**Keywords:** Chinese herbs, body fat mass, skeletal muscle, maximal oxygen consumption

## Abstract

*Rhodiola crenulata* (R) and *Cordyceps sinensis* (C) are commonly used herbs that promote health in traditional Chinese medicine. These two herbs have also been shown to exhibit anti-inflammation and antioxidant functions. Regular endurance training reveals potent endurance capacity, body composition improvement, and metabolic-related biomarker benefits. However, it is not known whether the combination of *Rhodiola crenulata* and *Cordyceps sinensis* (RC) supplementation during endurance training provides additive health benefits. The purpose of this study was to investigate the effects of 8-week endurance training plus RC supplementation on body composition, oxidative stress, and metabolic biomarkers in young sedentary adults. Methods: Fourteen young sedentary adults (8M/6F) participated in this double-blind randomized controlled study. Participants were assigned to exercise training with placebo groups (PLA, *n* = 7, 4M/3F; age: 21.4 ± 0.4 years) and exercise training with the RC group (RC, 20 mg/kg/day; *n* = 7, 4M/3F; age: 21.7 ± 0.4 years). Both groups received identical exercise training for eight weeks. The body composition, circulating oxidative stress, and blood metabolic biomarkers were measured before and after the 8-week intervention. Results: Improvement in body composition profiles were significantly greater in the RC group (body weight: *p* = 0.044, BMI: *p* = 0.003, upper extremity fat mass: *p* = 0.032, lower extremity muscle mass: *p* = 0.029, trunk fat mass: *p* = 0.011) compared to the PLA group after training. The blood lipid profile and systemic oxidative stress makers (thiobarbituric reactive substanceand total antioxidant capacity) did not differ between groups. Although endurance training markedly improved endurance capacity and glycemic control ability (i.e., fast blood glucose, insulin, and HOMA index), there were no differences in these variables between treatments. Conclusions: In this preliminary investigation, we demonstrated that an 8-week RC supplementation (20 mg/kg/day) faintly enhanced endurance training-induced positive adaptations in body composition in young sedentary individuals, whereas the blood lipid profile and systemic oxidative stress states were not altered after such intervention.

## 1. Introduction

Physical inactivity leads to adverse health effects including muscle loss, weight and fat gain, low-grade systemic inflammation, higher oxidative stress, and increased risk of developing type 2 diabetes, cardiovascular diseases, and certain cancers [1,2,3,4,5]. These chronic metabolic disorders are known to initiate at younger ages and gradually develop over decades until the clinical diseases emerge [6]. Although endurance exercise shows multiple benefits against dyslipidemia [7], obesity development [8], oxidative damage [9], metabolic disorders [10], and future cardiovascular events [11], endurance training-induced improvements are not exhibited in all of the above aspects. For example, Meredith and colleagues reported that 12-weeks of endurance training at 70% peak O_2_ consumption had no effect on body weight or body composition in young and elderly healthy adults [12]. Meanwhile, Kitaoka and co-workers found that although endurance training enhanced insulin sensitivity in both skeletal muscle and adipose tissues in young, normal-weight Japanese women, exercise training did not influence their fasting insulin/glucose levels, total triglycerides, LDL-C (low-density lipoprotein cholesterol), and hsCRP (high sensitivity C-reactive protein) [13]. Additionally, another study reported that 8-weeks of exercise training significantly increased aerobic capacity, but the endogenous antioxidant status (SOD, catalase, and GSH) were not markedly changed in healthy sedentary individuals [14]. Using Chinese herbal ingredients to maintain or improve human health has been practiced for hundreds of years. Chinese herbs are widely sought after for their biological properties including anti-diabetes, anti-oxidant, anti-inflammation anti-allergy, vasodilatory, anti-fatigue, anti-cancer, and cardio-protection [15,16,17,18,19,20]. The integration of innovative herbal-based dietary supplements with effective programmed endurance training for improved performance is needed.

*Rhodiola crenulata*, one of the Crassulaceae family, is a traditional herb that has been widely investigated for its bioactive compounds and therapeutic benefits. *Rhodiola crenulata* is traditionally used for treating acute mountain sickness, lung disorders, burns, soft tissue injuries, and diabetes as well as improving physical endurance and sleep quality [21]. Most of the above effects are attributed to salidroside, *p*-tyrosol, and rosavins. The major property of these bio-compounds is their antioxidant capacity [22,23]. A previous study showed that long-term *Rhodiola crenulata* supplementation significantly reduced blood glucose level, improved the liver and type 2 diabetes [17]. Another study indicated that this herb decreased plasma insulin, triglycerides, and HOMA-IR value levels in an animal model with metabolic syndrome and type 2 diabetes [21], suggesting that *Rhodiola* supplementation could be used as an effective strategy to improve metabolic derangements. *Cordyceps sinensis*, also called Chinese caterpillar fungus, is one of the most valuable Chinese traditional herbs. *Cordyceps sinensis* supplementation has been shown to regulate blood pressure by stimulating vessel dilation via nitric oxide release [18]. This increases tissue oxygen exchange [18], improves the lipid profile [19], and enhances endurance performance [24]. *Cordyceps sinensis* extract supplementation for two weeks appears to increase heart rate variability and muscle anti-fatigue capacity during exhaustive running testing on sedentary individuals [20]. This suggests that *Cordyceps sinensis* exerts an ergonomic effect on human health and performance. In line with the ergogenic effects of *Rhodiola crenulata* and *Cordyceps sinensis*, whether combining these two herbs could further promote human health and exercise performance is not well understood. 

To our knowledge, only two studies have examined a combination of *Rhodiola* and *Cordyceps* on exercise performance and muscle oxygen saturation in athletes. Our group previously reported that 2-weeks of *Rhodiola crenulata* and *Cordyceps sinensis* supplementation (2000 mg/day) markedly alleviated the physiological stress induced by high altitude training and further improved aerobic performance [25]. Colon and colleagues investigated the short-term *Cordyceps sinensis* and *Rhodiola rosea* supplementation (2000 mg/day) effects on muscle tissue oxygen saturation in male cyclists during maximal exercise. They reported that this supplementation did not markedly increase muscle oxygen saturation [26]. However, it is still unclear whether long-term administration of a *Rhodiola crenulata* and *Cordyceps sinensis* mixture is capable of improving health-related variables in young sedentary individuals. 

With this in mind, we hypothesized that regular endurance training plus *Rhodiola crenulata* and *Cordyceps sinensis* mixture (RC) supplementation could generate increased improvement in health-related variables in young sedentary individuals. The purpose of this study was therefore to determine the effect of 8-weeks of endurance training plus RC supplementation on body composition, oxidative stress, and metabolic biomarkers in young sedentary adults.

## 2. Materials and Methods

### 2.1. Participants

Fourteen young sedentary individuals (male: 8; female: 6) were recruited in this study. Participants with cardiovascular disease, diabetes, musculoskeletal disorders, or inability to perform regular endurance exercise were excluded. Participants were stratified using body weight, maximal oxygen uptake, and gender and assigned into two groups: exercise training with the placebo group (placebo, *n* = 7, four male and three female, age: 21.4 ± 0.4 years) and exercise training with *Rhodiola crenulata* and *Cordyceps sinensis* supplementation group (RC, *n* = 7, four male and three female, age: 21.7 ± 0.4 years). All participants were provided a written informed consent after careful explanation of the purpose, procedures, and possible potential risks in this study. This study was approved by the Institute Review Board (IRB) of Fu Jen Catholic University (FJU-IRB NO: C103054).

### 2.2. Experimental Design

**Figure 1:**
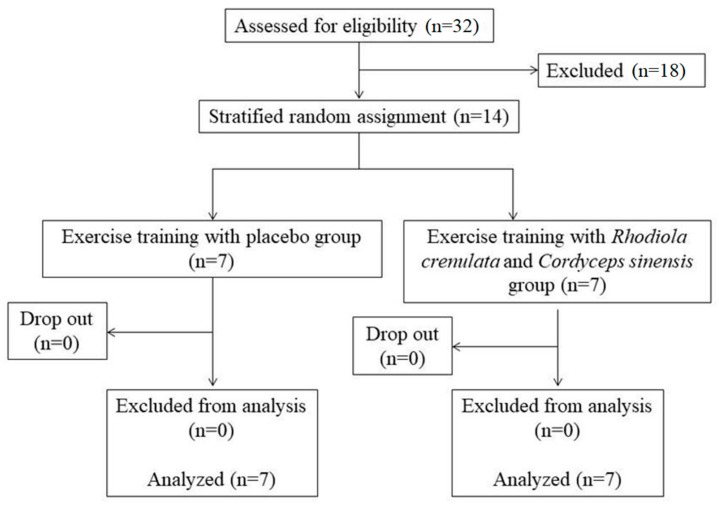
Flow chart of the study.

A randomized, double-blind, placebo-controlled experimental design was used in this study. One week prior to the intervention, the participants’ baseline parameters and maximal oxygen consumption (VO_2_max) were measured to determine the individual exercise training intensity. This study was conducted between April to June in 2017. All participants were randomly assigned to follow two groups: placebo (*n* = 7) and RC (*Rhodiola crenulata* and *Cordyceps sinensis*, *n* = 7) based on their basic characteristics (i.e., body weight, VO_2_max and gender). Participants allocated to the RC group were supplemented with RC (20 mg/kg/day; ranged 1060–1800 mg/day; ratio of *Rhodiola*: *Cordyceps* = 6:4; location/year of *Rhodiola* harvest: Tibet, China/2014; location/year of *Cordyceps* harvest: Qinghai, China/2014; TCM Biotechnology International Corp, Taipei, Taiwan) after breakfast, five days a week for eight weeks. To ensure the consistency, the same lot of supplements was provided during the entire study period. The placebo group consumed a placebo (no calorie sweetener, Sentosa, Taiwan) in the same way as the RC group. To ensure blind masking, the placebo capsules were provided by a blind research team using capsules with the same appearance and administration route as the active RC capsules. Both groups participated in identical endurance exercise training (three days/week) with gradually increased exercise intensity and duration during the eight weeks. A flow chart of this study is shown in Figure 1. During the eight weeks of intervention, all participants were asked to maintain their dietary pattern to diminish the potential for confounding factors. Participants were also asked to report via phone community software (LINE, LINE Corporation, Tokyo, Japan) after completing either the placebo or RC supplementation.

### 2.3. Maximal Oxygen Uptake (VO_2_max)

Maximum oxygen uptake was measured using the incremental load protocol after the fasting blood sample was collected. The VO_2_max test was performed on a cycle ergometer (Corival, Lode, Netherlands). The initial warm-up load was set at 45 watts for 5 minutes and then progressively increased by 15 watts every minute with 75 rpm pedal to exhaustion. The heart rate was measured using a wireless heart rate monitor (Polar RS800CX, Polar Electro, Kempele, Finland). The following criteria were used to determine the VO_2_max during the test: (1) heart rate ≥ HRmax ± 10%; (2) blood lactate value ≥ 10 mmol/L; 3() rating of perceived exertion (RPE) ≥ 17; and (4) pedal per minute ≤ 60 rpm. When the participant reached at least two of the above criteria, the time and watts at exhaustion were recorded and the VO_2_max value calculated using the following formula: VO_2_max = 10.51 * exhausted watt + 6.35 * body weight (kg) − 10.49* age + 519.3 mL/min [27]. Once the predicted VO_2_ max was calculated, the maximal work rate (Wmax) was calculated using the following formula to determine the training intensity: Watts at the target intensity = [(VO_2_max mL * target percentage) − 300]/12.5. 

### 2.4. Major Chemical Compound Analyses of Rhodiola/Cordyceps

The RC product used in this study was manufactured by TCM Biotechnology International Corp, Taiwan. The commercialized RC supplement was manufactured/packed and quality analyses were performed in autumn 2015. The expiration period for this product was 3 years after manufacturing and packing, and the RC supplements used in our human trial were all provided in prior to the expiration date to ensure the bio-stability (human trial experiment period was conducted between late spring to early summer in 2017). In addition, all used supplements were stored in the condition suggested by the manufacturer. Based on the quality analyses, the stability of primary functional ingredients between lots are shown as follows (difference between lots: salidroside = 0.17%; uridine = 0.16%; guanosine = 0.03%; adenosine = 0.02%; polysaccharide = 1.53%; the lots for current study were analyzed in autumn 2015, and the compared lots were analyzed in spring 2016). The major RC chemical compounds were analyzed using high-performance liquid chromatography (HPLC) and alcohol precipitation according to the individual compound chemical properties. 

**Figure 2:**
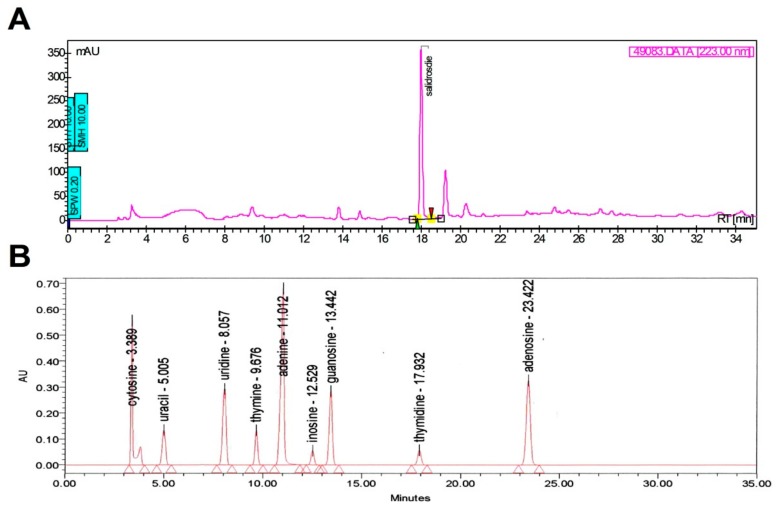
Chromatographic fingerprints of the *Rhodiola* (**A**) and *Cordyceps* (**B**) supplement by high-performance liquid chromatography (HPLC) analysis.

According to the results of the chemical analyses of the current using supplements (*Rhodiola*, Lot no. FD09023; *Cordyceps*, Lot no. 10T121-TM; analyses were performed in autumn 2015), the major bioactive functional compound in *Rhodiola* was salidroside (14.27 mg/g, 1.43%), and the bioactive compounds in *Cordyceps* were uridine (3.07 mg/g, 0.307%), guanosine (4.91 mg/g, 0.491%), adenosine (3.89 mg/g, 0.389%), and polysaccharide (94 mg/g, 10.25%). Figure 2 presents the obtained chemical analyses results for *Rhodiola* (2A) and *Cordyceps* (2B).

### 2.5. Eight-Week Training Program

The ergometer training protocol was performed three days/week for eight weeks. The initial exercise intensity was set as 60% Wmax and gradually increased to 75% Wmax. The exercise duration was gradually increased from 30 min to 60 min, as seen in Table 1. During training, heart rate, and power output were monitored to ensure the participants’ physical status and exercise intensity. All training processes were supervised by professional trainers to ensure the training quality and consistency.

### 2.6. Anthropometric Measurements

All participants were informed to not perform any type of exercise three days before the measurement. Blood pressure was measured in the sitting position after 5 minutes rest using a digital sphygmomanometer (Microlife BP3AQ1, Microlife AG, Widnau, Switzerland). Participant height was measured using a stadiometer (Dong Sahn Jenix Co. Ltd., Seoul, Korea). The body composition (body mass, fat mass, muscle mass) was measured using the bioelectrical impedance analysis (BIA) system InBody 230 (Biospace Co., Ltd, Seoul, Korea) after 10 hours of overnight fasting. To ensure the accuracy and precision of the body parameter measurements, we verified the consistency of our current using the BIA instrument through repeatedly measuring a health young individual for eight tests within one hour. According to the verification data, the intra CV% in different parts of the body were obtained and ranged from 0.0–3.4% (muscle mass measurement intra-CV% = 0.3–1.0%; adipose tissue measurement intra-CV% = 0–3.4%), which provided us with strong confidence in using the BIA instrument to evaluate the primary outcomes in this study. In brief, the participants were asked to wear similar light clothing to ensure consistency for both the pre-and-post tests. To ensure stability for the BIA measurements, the participants were measured in the upright steady standing position with their bare feet positioned on the footpad electrodes and their hands on the handle electrodes. During the BIA measurement, a small electrical current was transmitted through the body. The resistance was recorded and total body water and the percentage of fat mass were calculated using the built-in InBody 230 system software. Additionally, to eliminate possible confounders, the drinking of water and all liquids containing caffeine were prohibited, and all wearable metal items were asked to be removed before the body composition measurement. 

### 2.7. Hematological Analyses

All participants were instructed not to use alcohol, medicine, or other nutritional supplements for one week and to not perform any type of exercise for three days before the blood collection. Fasting venous blood samples (5 mL) were collected in the morning (8:00 am) after ten hours of overnight fasting. The blood lipid profile (triglyceride, total cholesterol, LDL-cholesterol, and HDL-cholesterol) and glucose were used on UniCel DxC-800 Synchron Clinical Systems (Beckman Coulter, Inc., Brea, CA, USA) for analysis. The serum insulin concentration was evaluated using the INS-IRMA KIP1251-KIP1254 kit (BioSource, Nivells-Belgium) according to the radio-immuno assay method. PerkinElmer Automatic Gamma Counters (1470 Wizard Series, PerkinElmer Automatic, Santa Clara, CA, USA) were used to determine the insulin level. 

### 2.8. TBARS and TAC Analyses

Plasma thiobarbituric reactive substance (TBARS) and total antioxidant capacity (TAC) were measured using a commercial kit (TBARS and TAC assay, Caymen chemical, Ann Arbor, IM, USA) in accordance with the kit instructions. The TBARS concentration read the absorbance at 540 nm in the enzyme linked immunoassay (ELISA) reader (infinite M200PRO, TECAN, Salzburg, Australia). The plasma TAC level was read under 750 nm absorbance in the same ELISA reader. 

### 2.9. Statistical Analysis

All data were analyzed using SPSS 22.0 software (SPSS, Chicago, IL, USA). Data are presented as mean ± standard error of the mean (mean ± S.E.M). All data used the Shapiro–Wilk test to examine the distribution normality before statistical analysis. Differences in the baseline and changes (Post-Pre) between groups were analyzed using the independent t-test. The paired t-test was used to determine the differences between the baseline and post eight-week intervention. Statistical significance was set at *p <* 0.05.

## 3. Results

### 3.1. Characteristics of Participants

The participants’ characteristics are presented in Table 2. All participants were assigned into the placebo and RC groups according to the baseline body mass, maximal oxygen uptake, and gender. There were no significant differences between the two groups in gender, age (21.4 ± 0.4 vs. 21.7 ± 0.4 years), body weight (69.7 ± 5.0 vs. 67.6 ± 4.8 kg), height (168.0 ± 2.3 vs. 167.7 ± 4.0 cm) and VO_2_max (36.9 ± 2.2 vs. 35.4 ± 1.1 ml/kg/min) before intervention.

### 3.2. Body Composition Assessment

**Figure 3:**
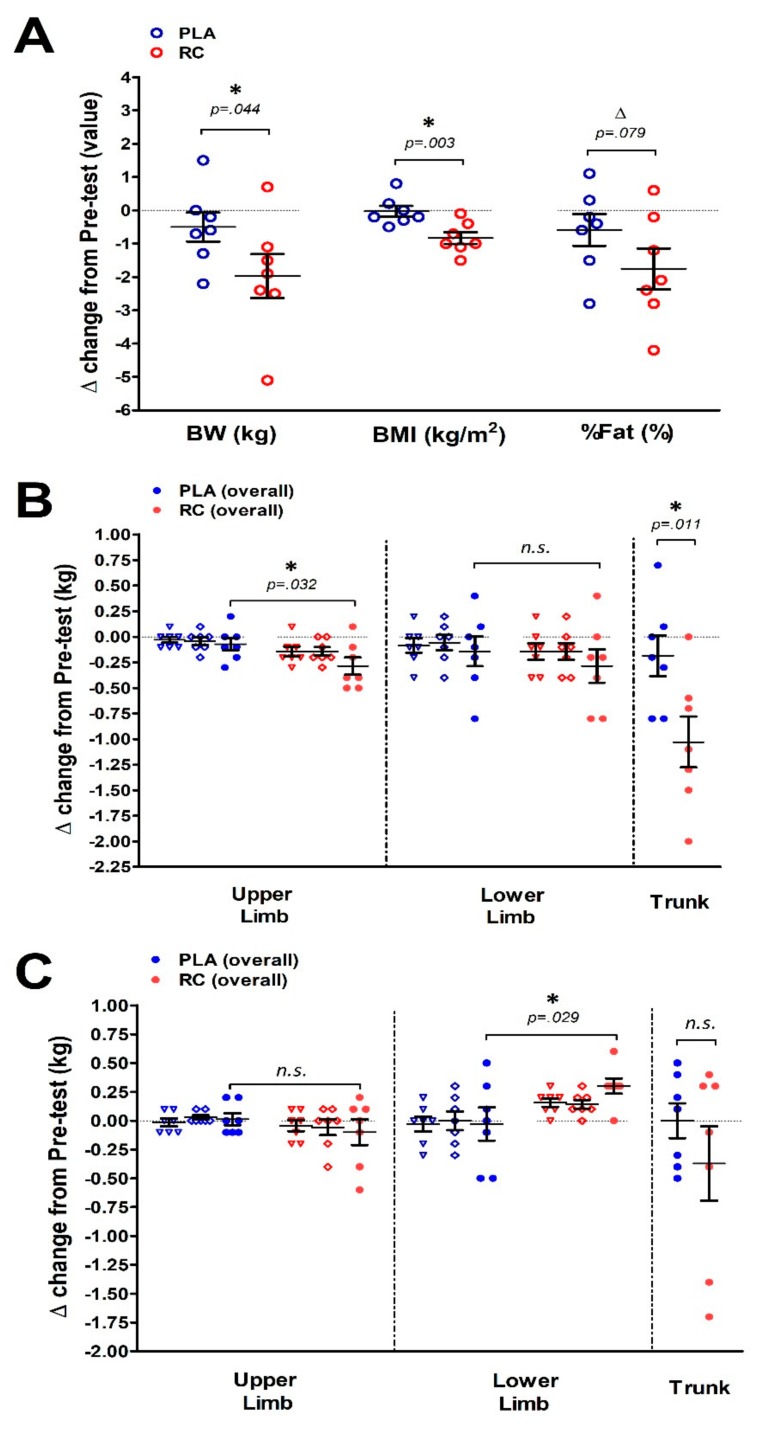
Changes in the body composition between the placebo and RC groups after eight weeks of intervention. The RC group showed lower body weight, BMI (**A**) and upper limb adipose tissue (**B**) after intervention. Compared to the placebo group, the RC group showed higher muscle mass in the lower limb (**C**) after treatment. BW: Body weight; BMI: Body mass index; % BF: Percentage of fat mass. Open blue circle: PLA group; Open red circle: RC group (Figure 3A). Blue open triangle/diamond: Left/right arm and leg in PLA; Blue solid circle: Upper limb/Lower limb (sum of left + right arm/leg in PLA). Red open triangle/diamond: Left/right arm and leg in RC; Red solid circle: Upper limb/Lower limb (sum of left + right arm/leg in RC) (Figure 3B,C). * denotes the significant difference between the placebo and RC groups (*p* < 0.05). Values are expressed as the mean ± S.E.M.

Table 3 shows the body composition assessments. There were no significant differences in body weight, body mass index (BMI), percentage of body fat, skeletal muscle tissues (included upper and lower limbs, and trunk), and adipose tissues (included upper and lower limbs, and trunk) between groups at Pre-test. However, compared to the Pre-test, the body weight, BMI, and percentage of body fat, upper limbs fat mass and trunk fat mass were significantly lower than the Post-test only in the RC group after eight weeks of intervention. In order to further compare the effects of providing RC supplementation after eight weeks of exercise training, we calculated the change values (Post–Pre) to reflect the degree of improvement in body composition parameters (Figure 3). RC supplementation generated significant improvement in body weight reduction, BMI (Figure 3A), and upper limb adipose tissue (Figure 3B) compared to the placebo group following eight weeks of treatment. We also observed that RC supplementation markedly increased the lower limb muscle mass (Figure 3C) after the intervention.

### 3.3. VO_2_max, Metabolic Parameters and Oxidative Stress Markers Assessment

**Figure 4:**
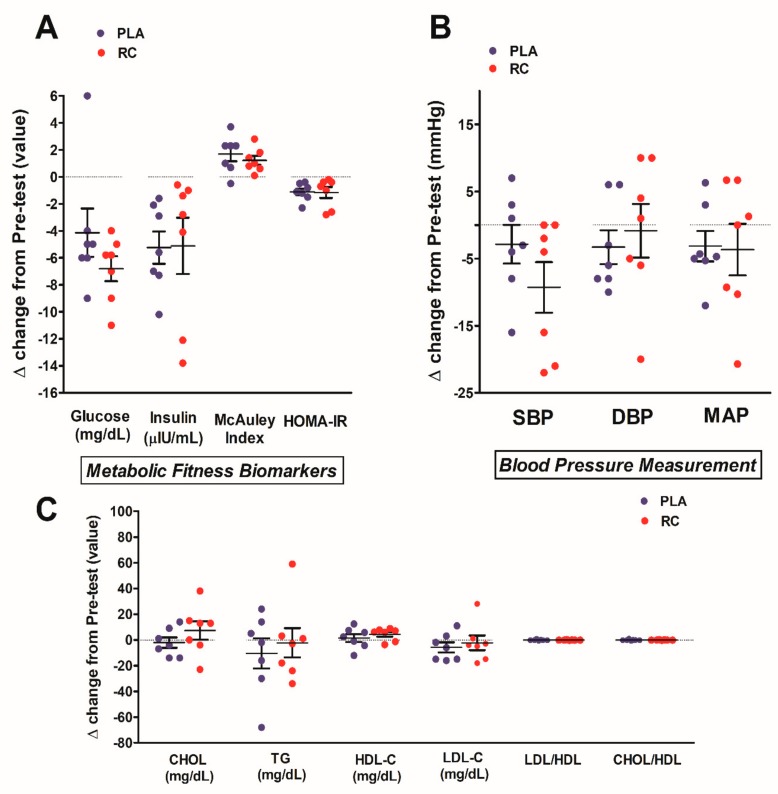
Changes in the metabolic parameters between the placebo and RC groups following eight weeks of intervention. No difference in the glucose metabolism (**A**), blood pressure (**B**), and lipid profile (**C**) changes in both groups after treatment. Blue solid circle: placebo (PLA); Red solid circle: RC supplementation (RC). SBP: Systolic blood pressure, DBP: Diastolic blood pressure. MAP: Mean arterial pressure. CHOL: Cholesterol, TG: Triglyceride, HDL-C: High-density lipoprotein cholesterol, LDL-C: Low-density lipoprotein cholesterol, LDL/HDL: LDL-C to HDL-C ratio. Values are expressed as the mean ± S.E.M.

**Figure 5:**
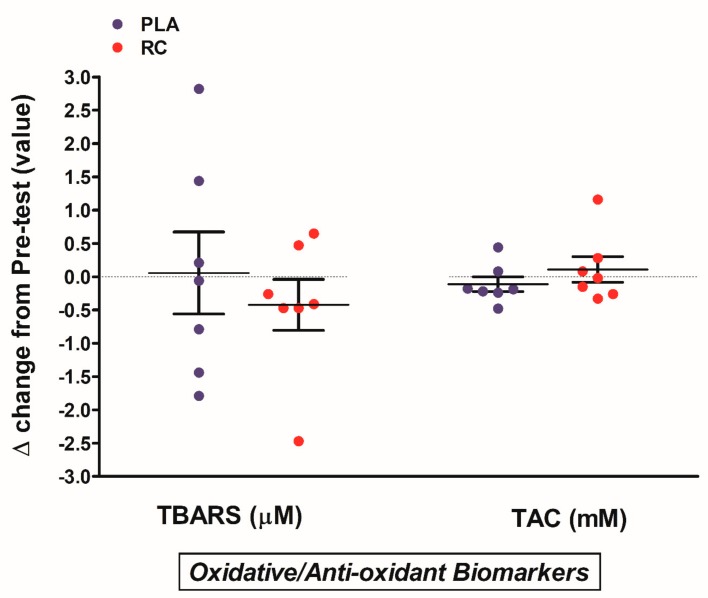
Changes in the oxidative and anti-oxidative biomarkers between the placebo and RC groups after eight-week intervention. No significant difference in TBARS and TAC levels between groups after intervention. Blue solid circle: placebo (PLA); Red solid circle: RC supplementation (RC). TBARS: Thiobarbituric acid reactive substances, TAC: Total antioxidant capacity. Values are expressed as Mean ± S.E.M.

The VO_2_max, metabolic parameters, and oxidative stress markers are displayed in Table 4. After eight weeks of intervention, VO_2_max was markedly enhanced in both groups; furthermore, the levels of glucose, insulin, and HOMA-IR were significantly decreased in both the placebo and RC groups. The diastolic blood pressure, systolic pressure, and mean arterial pressure showed no significant differences in both groups at pre and post-intervention. There were no significant changes in total cholesterol (CHOL), triglyceride (TG), high-intensity lipoprotein (HDL-C), low-intensity lipoprotein (LDL-C), LDL-C-to-HDL-C ratio (LDL/HDL), and CHOL-to-HDL-C ratio (CHOL/HDL) in both groups. We did not observe a marked change in TBARS and TAC levels in both groups after eight weeks of intervention. Figure 4 shows the degree of improvement in the metabolic and oxidative stress parameters after eight weeks of intervention. There were no significant differences in the changes in glucose, insulin, McAuley index, and HOMA-IR in both groups after treatment (Figure 4A). The changes in blood pressure are displayed in Figure 4B. There were no significant changes in systolic blood pressure, diastolic blood pressure, and mean arterial pressure between the placebo and RC groups. The changes in lipid profile are shown in Figure 4C. The changes in blood CHOL, TG, HDL-C, LDL-C, LDL/HDL ratio, and CHOL/HDL ratio showed no significant differences in both groups after eight weeks of intervention. The changes in TBARS and TAC showed no marked differences between the two groups after treatment (Figure 5).

## 4. Discussion

To our knowledge, this study is the first long-term study to investigate the endurance exercise training plus anti-oxidant supplementation effects using *Rhodiola/Cordyceps* (RC)-based herbal supplement on metabolic-related health biomarkers in young sedentary adults. Eight weeks of endurance exercise training was effective in this study in increasing aerobic endurance and glycemic control capacity. Of interest, we observed that the combination of endurance training plus RC intervention further ameliorated body composition (i.e.,↓ body weight, ↓ fat mass in the upper extremity and trunk, ↑muscle mass in the lower extremity). However, there were no changes in blood lipid profile, oxidative stress state, and total antioxidant capacity in both training alone and training plus RC groups. According to our present preliminary findings, in combination with the eight-week endurance training program, RC supplementation did not change the oxidative stress/antioxidant capacity status, but did elicit certain degrees of improvements in body composition in these young sedentary individuals. For better training adaptations, the combination of RC and endurance exercise training may show the possible benefits of combined intervention through positive changes in body composition in this young inactive population.

Although there have been numerous studies investigating the independent ergogenic effects of *Rhodiola* [28,29,30,31,32,33] and *Cordyceps* [34,35,36,37] in the literature, the results obtained here are still controversial among studies using varied experimental models. Short-term *Rhodiola* extracts (600–1500 mg/day for 3–30 days) have been demonstrated to exhibit ergogenic benefits among varied experimental models [28,29,30]. On the other hand, few studies have reported the positive ergogenic benefits of *Cordyceps* (1000–4000 mg/day for 21 days to 12 weeks) on enhancing exercise performance in both animal and human models [34,35,36,37]. These findings thus indicate the possibility of combining these two herbal-based ingredients to improve exercise adaptations. Still, several previous studies have examined the *Rhodiola* alone for 4–30 days (dose: 170–1500 mg/day) effects on exercise performance [31,32,33]. These studies reported that short-term *Rhodiola* ingestion failed to further improve aerobic capacity and intramuscular ATP turnover in well-trained individuals and physically active youth [31,32,33]. There have only been two human studies using the combination of *Rhodiola* and *Cordyceps* on exercise performance in athletes [25,26] that lead to the little available knowledge on the effectiveness of applying these herbal-based mixtures during exercise training in general populations.

After intensively reviewing the related literature, no earlier studies, to our knowledge, have been conducted to determine the benefits of using a *Rhodiola/Cordyceps* mixture during long-term exercise training (>6 weeks) on the metabolic health of exclusively young sedentary individuals. The three earlier human studies that used *Rhodiola* [31,32,33], and the current study, noted similarly that eight weeks of *Rhodiola*/*Cordyceps* mixture supplementation (20 mg/kg/day; ranging from 1060–1800 mg/day; ratio of *Rhodiola:Cordyceps* = 6:4) was unable to further promote exercise-induced endurance performance in these sedentary young adults (Table 4). However, there are several inconsistent findings. For example, acute *Rhodiola* administration has been previously reported to ameliorate anaerobic performance in active females (1500 mg/day for three days) [30]. *Cordyceps sinensis* (1000 mg/day) for 12-weeks improved exercise performance in healthy older subjects [35]. Additionally, a two week ingestion of R/C-based supplement (R: 1400 mg + C: 600 mg per day) was shown to boost aerobic performance after short-term high altitude training in highly trained athletes [25]. Among the blood lipid profile parameters, although there were slight but non-significant increases in total cholesterol level in the RC group (Figure 4C), the cholesterol levels of both groups were still within the clinical normal range (<200 mg/dL) at this age. This discrepancy could be due to the varied dosage of *Rhodiola* and *Cordyceps*, duration of intervention (three days to 12 weeks), characteristics of the participants (age, genders, physical activity levels, etc.), the category of outcome measurements (e.g., endurance capacity vs. anaerobic performance), and training environment (e.g., sea level vs. high-altitude). 

Aerobic training may lead to increased antioxidant activity in animal and human models [38,39,40]. Previous studies have also indicated the antioxidant enzyme activity in trained individuals was markedly higher than in sedentary ones [41]. However, the benefits of aerobic training on improving the antioxidant capacity in humans are controversial. Tiidus et al. (1996) investigated the effects of eight weeks of endurance training (35 min/3 times/wk) on vastus lateralis muscle antioxidant status. The results reported a significant increase in aerobic capacity and muscle citrate synthase activity after training, whereas the endogenous antioxidant status (SOD, catalase, and GSH) were unaltered in healthy sedentary adults after training [14]. Bergholm et al. reported that antioxidant circulations (SH-groups, α-tocopherol, β-carotene, retinol) were significantly decreased in fit male individuals following endurance training [42]. In line with the above studies, we did not observe significant changes in circulating TBARS and TAC after eight weeks of endurance training. These results indicate that the improvement in endogenous antioxidant status after endurance training might be affected by different training modes and individual characteristics. 

*Rhodiola* and *Cordyceps* supplements have been documented to enhance antioxidant capacity via reducing free radical production as well as enhancing singlet oxygen and hydrogen peroxide scavenging [36,43,44,45]. However, fewer studies have investigated the benefits of RC supplementation with endurance training on antioxidant capacity in young sedentary adults. Parisi and colleagues investigated the effects of four week chronic *Rhodiola* supplementation (170 mg/day) in 14 trained male athletes. The results showed that this herbal supplement did not affect the blood antioxidant status and inflammatory parameters. The proposed mechanism might be related to the polyphenol content in *Rhodiola* [31]. Chen et al indicated that *Rhodiola* had higher polyphenol content, which presented higher potential ability in singlet oxygen and hydrogen peroxide scavenging [45]. Consistent with the finding by Parisi et al., we did not observe marked changes in all endogenous antioxidant parameters (TBARS and TAC). Chronic RC supplementation seemingly did not affect circulating redox homeostasis in these young adults even if they underwent regular endurance training. 

Regular exercise training has been proven to markedly reduce body weight and body fat in overweight and obese individuals without accompanying dietary restrictions [46,47,48]. However, in this study, we found that the placebo group did not exhibit improved body composition even though the subjects underwent eight week endurance training. This inconsistent result was possibly due to the different training duration and intensity. Tremblay and colleagues reported that 20-week ergocycle training (intensity: 60% increased to 80% HR; duration: 30 increased to 45 min; frequency: four times increased to five times per week) did not generate effective body weight and body fatness improvement compared to 15-week high intensity intermittent training in young healthy adults when the energy cost of training was corrected [49]. Note that the exercise training program in this study was similar to that of Tremblay et al., which indicated that the exercise training intensity could be the major factor that influenced the body composition effects in these young sedentary participants. Interestingly, a key finding in the current study was that the RC supplement was capable of decreasing the body weight, upper extremity fat mass, and trunk fat mass after eight weeks of endurance training. Until now, the underlying RC supplement benefit mechanisms on body composition after endurance training were not fully understood. Lin and colleagues investigated the *Rhodiola Crenulata* extract (RCE) effects on hepatic lipid metabolism. The related mechanisms underlying such effects reported that RCE at concentrations of 1.5, 3.0, 15.0, and 30.0 μg/mL significantly suppressed hepatic lipogenesis by increasing the AMP-activated protein kinase (AMPK) activation and fatty acid oxidation. In addition, RCE also inhibited protein and gene expression in lipogenesis-related genes (SREBP-1c, C/EBP, and FAS) in the rat liver [50]. Pomari and coworkers demonstrated 3% salidroside (RS) or 1% salidroside and 3% rosavine (RR) extracts showed a significant increase in GATA3, WNT3A, and WNT10B gene expressions, which are involved in adipogenesis inhibition as well as a decrease in SLC2A4 and FGF2 gene expressions, involved in adipocyte function in human visceral pre-adipocytes during differentiation [51]. Together with the existing evidence, our current findings suggest that the beneficial effects of RC supplementation on reducing body weight and body fatness might be due to increasing fat oxidation and adipogenesis inhibition from *Rhodiola* during intervention. 

A greater increase in muscle mass was observed in the group treated with exercise training plus RC supplementation. Although there is a lack of direct human research evidence on the benefits of either *Rhodiola* and *Cordyceps* on muscle mass, several lines of cell-culture and animal evidence may provide, at least in part, an explanation for our current findings. Salidroside, one of the primary functional ingredients of *Rhodiola*, has been reported to preserve muscle mass in the mouse cancer-cachexia model via activating mammalian rapamycin signaling (mTOR) [52] and prevent the stress-induced repression of mTOR signaling [53]. Furthermore, the *Cordyceps* fruit body extract is capable of activating protein kinase B (AKT)/mammalian rapamycin (mTOR) target pathways in rodent tissue [54]. In considering the key role of mTOR in promoting muscle growth, we speculated that the positive changes in muscle mass in endurance training with RC supplementation might be due to the potential effects of *Rhodiola* and *Cordyceps* on improving muscle hypertrophy. In supporting previous findings based on animal and cell-culture studies, we further provided human-based evidence on the potential benefits of RC supplementation in improving fat loss and muscle growth during endurance training. However, we also observed that the upper limbs and trunk muscle masses were slightly lower in the RC group after intervention, even though no statistical differences were found. One possible explanation could be the exercise type (i.e., cycling) used in this study, and the much lower involvement degree of both the trunk and upper limbs during exercise compared with the lower limbs. Additionally, *Rhodiola* and *Cordyceps* have both been reported to exert the ability to activate both catabolic (AMPK) and anabolic (mTOR) signaling systems in varied tissues [50,53,54]. It has not been clear whether certain interactions between both signaling systems in response to RC plus such endurance training intervention would alter muscle mass changes in relatively less-recruited muscles. 

Several recent studies have revealed that the human body seems to be able to tolerate a limited increase in free radicals. A bursting increase in reactive oxygen species (ROS; free radicals) is needed for exercise adaptations to occur during intensified training [55]. Several investigations also showed that chronic antioxidant treatment could hamper the physiological adaptions obtained from exercise training due to quenching oxidative stress-mediated adaptation signals [56,57,58,59]. This implies the possibility that long-term antioxidant supplementation might perturb and counteract training outcomes. *Rhodiola* and its primary functional ingredient, salidroside, have been demonstrated as effective antioxidants [60]. The three primary functional ingredients of *Cordyceps* including guanosine [61], adenosine [62,63], and polysaccharides [64] also exhibit clear antioxidant properties. Although both *Rhodiola* and *Cordyceps* have been proven to have considerable antioxidant benefits in varied experimental models, it has not been investigated as to whether long-term RC supplementation would negatively influence exercise adaptations (e.g., maximal oxygen uptake, glycemic control capacity, body compositions, etc.). Of interest, according to our present observations, using long-term RC (daily dose ranged from 1060–1800 mg, providing at 20 mg/kg body weight) during the eight week intervention should not perturb endurance training adaption. However, RC administration can still elicit profound benefits by further suppressing fat accumulation and lower extremity muscle gain during exercise training.

Here, we demonstrated that the provision of RC-based herbal supplementation (20 mg/kg) showed faintly positive benefits by increasing muscle mass and reducing body fat after eight weeks of endurance training in young sedentary adults. However, there were no substantial changes in antioxidant capacity and metabolic health profile following intervention. Note that a research crew periodically reminded all participants to maintain their physical activity level plus clear dietary guidance to ensure consistence and compliance. These preliminary results provide evidence that RC supplementation for a sedentary population further promotes certain training adaptations (i.e., ↑ muscle mass and ↓ fat mass). For the sedentary populations, although the participants involved in this study seems unlikely to spontaneously adopt an intensive exercise regime, their motivation to adhere in the training may be boosted with more substantial training outcomes through utilizing effective nutritional aids. Our findings, therefore, suggest a potential nutrient approach of using RC-based herbal supplementation to further enhance endurance training adaptations in individuals who intend to initiate their training program. However, the precise underlying mechanism(s) by which RC supplementation induces fat mass loss during exercise training warrants further investigation, meanwhile the future study with longer intervention would be needed to pursue the possible long-term effects. One limitation of this study is the relatively small sample size due to the difficulty in recruiting previously physical inactive young adults (several participants were excluded due to the inability to match the training arrangement), and this could possibly increase the chance for type 2 errors. Consequently, a larger sample size study might be needed in the future. 

## 5. Conclusions

In the current preliminary investigation, we demonstrated that eight weeks of herbal-based supplementation containing *Rhodiola* and *Cordyceps* (20 mg/kg/day; ratio of *Rhodiola:Cordyceps* = 6:4) faintly elicited positive changes in endurance training adaptations on body composition (i.e., ↓ body fat and ↑ muscle mass), but not on aerobic performance in physically inactive young adults. The blood lipid profile, systemic oxidative stress state, and total antioxidant capacity seemed to be unchanged after both interventions in this population. A future study with larger participants is warranted to further investigate the possible underlying mechanisms due to the relatively small sample size in this study.

## Figures and Tables

**Table 1:** Eight week training program.

Week	Frequency (times/week)	Intensity (%Wmax)	Duration (min)
Week 1	3	60	30/40/50
Week 2	3	60	60
Week 3	3	65	30/40/50
Week 4	3	65	60
Week 5	3	70	30/40/50
Week 6	3	70	60
Week 7	3	75	30/40/50
Week 8	3	75	60

**Table 2:** Baseline characteristics of the participants.

Baseline Characteristics	PLA	RC
Gender (male: female)	4 : 3	4 : 3
Age (years)	21.4 ± 0.4	21.7 ± 0.4
Body weight (kg)	69.7 ± 5.0	67.6 ± 4.8
Height (cm)	168.0 ± 2.3	167.7 ± 4.0
VO_2_max (ml/kg/min)	36.9 ± 2.2	35.4 ± 1.1

Values are expressed as the mean ± S.E.M. VO_2_max: maximum oxygen uptake. PLA: placebo; RC: *Rhodiola crenulata* and *Cordyceps sinensis* supplementation.

**Table 3:** Body composition assessment.

Body Composition	PLA		RC	
Assessment	Pre	Post	Pre	Post
Body weight (kg)	69.7 ± 5.0	69.2 ± 5.4	67.6 ± 4.8	65.7 ± 4.8 ^†^
BMI (kg/m^2^)	24.6 ± 1.4	24.5 ± 1.5	23.9 ± 0.9	23.0 ± 2.3 ^†^
Body fat (%)	26.6 ± 2.8	26.0 ± 2.8	27.3 ± 2.8	25.5 ± 2.7 ^†^
Skeletal muscle tissue				
Upper limbs (kg)	5.4 ± 0.4	5.4 ± 0.4	5.3 ± 0.7	5.2 ± 0.7
Lower limbs (kg)	16.2 ± 1.0	16.3 ± 1.0	15.0 ± 1.6	15.3 ± 1.6 ^†^
Trunk (kg)	22.6 ± 1.2	22.6 ± 1.3	22.2 ± 2.3	21.9 ± 2.2
Adipose tissue				
Upper limbs (kg)	2.5 ± 0.5	2.4 ± 0.6	2.3 ± 0.3	2.0 ± 0.3 ^†^
Lower limbs (kg)	5.8 ± 0.8	5.6 ± 0.8	5.3 ± 0.4	5.0 ± 0.4
Trunk (kg)	9.5 ± 1.6	9.4 ± 1.7	9.3 ± 0.8	8.3 ± 0.9 ^†^

Values are expressed as the mean ± S.E.M. BMI: body mass index, † denotes the significant difference from the Pre value (*p* < 0.05). PLA: placebo; RC: *Rhodiola crenulata* and *Cordyceps sinensis* supplementation.

**Table 4:** The changes in VO_2_max and metabolic parameters and oxidative stress markers after eight weeks of intervention.

Metabolic and Oxidative Stress	PLA		RC	
Measurement	Pre	Post	Pre	Post
VO_2_max (mL/kg/min) Glucose (mg/dL)	36.9 ± 2.2 80.0 ± 3.3	40.2 ± 3.0^†^ 75.9 ± 2.9 ^†^	35.4 ± 1.1 80.1 ± 1.3	39.5 ± 1.7^†^ 73.3 ± 2.1 ^†^
Insulin (µIU/mL)	13.2 ± 1.7	7.9 ± 0.8 ^†^	13.8 ± 2.0	8.7 ± 0.9 ^†^
McAuley Index	7.5 ± 0.5	9.2 ± 0.8	7.6 ± 0.7	8.8 ± 0.6
HOMA-IR	2.6 ± 0.4	1.5 ± 0.2 ^†^	2.7 ± 0.4	1.6 ± 0.2 ^†^
SBP (mmHg)	112.9 ± 3.2	110.0 ± 4.9	119.7 ± 2.9	110.4 ± 5.5
DBP (mmHg)	70.4 ± 2.1	67.1 ± 3.0	69.0 ± 2.2	68.1 ± 3.6
MAP (mmHg)	84. 6 ± 1.9	81.4 ± 3.0	85.9 ± 1.8	82.2 ± 3.6
CHOL (mg/dL)	179.8 ± 10.6	177.7 ± 10.5	180.4 ± 7.6	187.8 ± 10.0
TG (mg/dL)	78.1 ± 15.0	67.7 ± 17.2	69.5 ± 9.1	67.2 ± 15.7
HDL-C (mg/dL)	55.3 ± 6.7	56.8 ± 6.5	55.5 ± 3.7	59.9 ± 4.9
LDL-C (mg/dL)	106.8 ± 8.6	101.1 ± 11.8	108.0 ± 4.9	105.7 ± 6.9
LDL/HDL	2.1 ± 0.3	2.0 ± 0.4	1.9 ± 0.1	1.8 ± 0.2
CHOL/HDL	3.4 ± 0.4	3.4 ± 0.5	3.3 ± 0.2	3.2 ± 0.2
TBARS (µM)	4.1 ± 0.4	4.2 ± 0.9	5.0 ± 0.5	4.6 ± 0.4
TAC (mM)	4.9 ± 0.5	4.8 ± 0.4	4.3 ± 0.5	4.3 ± 0.4

Values are expressed as the mean ± S.E.M. VO_2_max: Maximum oxygen uptake, SBP: Systolic blood pressure, DBP: Diastolic blood pressure, MAP: Mean arterial pressure, CHOL: Cholesterol, TG: Triglyceride, HDL-C: High-density lipoprotein cholesterol, LDL-C: Low-density lipoprotein cholesterol, LDL/HDL: LDL-C to HDL-C ratio, CHOL/HDL: Cholesterol to HDL-C ratio, TBARS: Thiobarbituric acid reactive substances, TAC: Total antioxidant capacity. † denotes the significant difference from Pre value (*p* < 0.05). PLA: placebo; RC: *Rhodiola crenulata* and *Cordyceps sinensis* supplementation.

## References

[1] Biswas A., Oh P.I., Faulkner G.E., Bajaj R.R., Silver M.A., Mitchell M.S., Alter D.A. (2015). Sedentary time and its association with risk for disease incidence, mortality, and hospitalization in adults: A systematic review and meta-analysis. Ann. Intern. Med..

[2] De Rezende L.F.M., Lopes M.R., Rey-Lopez J.P., Matsudo V.K.R., Luiz O.D.C. (2014). Sedentary Behavior and Health Outcomes: An Overview of Systematic Reviews. PLoS ONE.

[3] Owen N., Sparling P.B., Healy G.N., Dunstan D.W., Matthews C.E. (2010). Sedentary Behavior: Emerging Evidence for a New Health Risk. Mayo Clin. Proc..

[4] Wilmot E.G., Edwardson C.L., Achana F.A., Davies M.J., Gorely T., Gray L.J., Khunti K., Yates T., Biddle S.J.H. (2012). Sedentary time in adults and the association with diabetes, cardiovascular disease and death: Systematic review and meta-analysis. Diabetologia.

[5] Patterson R., McNamara E., Tainio M., De Sá T.H., Smith A.D., Sharp S.J., Edwards P., Woodcock J., Brage S., Wijndaele K. (2018). Sedentary behaviour and risk of all-cause, cardiovascular and cancer mortality, and incident type 2 diabetes: A systematic review and dose response meta-analysis. Eur. J. Epidemiol..

[6] Warburton D.E., Nicol C.W., Bredin S.S. (2006). Health benefits of physical activity: The evidence. Can. Med. Assoc. J..

[7] Durstine J.L., Grandjean P.W., Davis P.G., Ferguson M.A., Alderson N.L., DuBose K.D. (2001). Blood lipid and lipoprotein adaptations to exercise: A quantitative analysis. Sports Med..

[8] Williamson D.F., Madans J., Anda R.F., Kleinman J.C., Kahn H.S., Byers T. (1993). Recreational physical activity and ten-year weight change in a US national cohort. Int. J. Obes. Relat. Metab. Disord..

[9] Sallam N., Laher I. (2016). Exercise Modulates Oxidative Stress and Inflammation in Aging and Cardiovascular Diseases. Oxidative Med. Cell. Longev..

[10] Jeon C.Y., Lokken R.P., Hu F.B., Van Dam R.M. (2007). Physical Activity of Moderate Intensity and Risk of Type 2 Diabetes: A systematic review. Diabetes Care.

[11] Sesso H.D., Paffenbarger R.S., Lee I.M. (2000). Physical activity and coronary heart disease in men: The Harvard Alumni Health Study. Circulation.

[12] Meredith C.N., Frontera W.R., Fisher E.C., Hughes V.A., Herland J.C., Edwards J., Evans W.J. (1989). Peripheral effects of endurance training in young and old subjects. J. Appl. Physiol..

[13] Kitaoka K., Takeuchi M., Tsuboi A., Minato S., Kurata M., Tanaka S., Kazumi T., Fukuo K. (2017). Increased Adipose and Muscle Insulin Sensitivity without Changes in Serum Adiponectin in Young Female Collegiate Athletes. Metab. Syndr. Relat. Disord..

[14] Tiidus P.M., Pushkarenko J., Houston M.E. (1996). Lack of antioxidant adaptation to short-term aerobic training in human muscle. Am. J. Physiol. Integr. Comp. Physiol..

[15] Cui J.L., Guo T.T., Ren Z.X., Zhang N.S., Wang M.L. (2015). Diversity and antioxidant activity of culturable endophytic fungi from alpine plants of *Rhodiola crenulata*, *R. angusta*, and *R. sachalinensis*. PLoS ONE.

[16] Yang X., He T., Han S., Zhang X., Sun Y., Xing Y., Shang H. (2019). The Role of Traditional Chinese Medicine in the Regulation of Oxidative Stress in Treating Coronary Heart Disease. Oxidative Med. Cell. Longev..

[17] Fan Y., Tan R., Jiang L. (2007). Evaluation of treatment of 27 cases of type 2 diabetic patients with Rhodiola crenulata tea. Chin. J. Mod. Appl. Pharm..

[18] Kan W.C., Wang H.Y., Chien C.C., Li S.L., Chen Y.C., Chang L.H., Cheng C.H., Tsai W.C., Hwang J.C., Su S.B. (2012). Effects of Extract from Solid-State Fermented *Cordyceps sinensis* on Type 2 Diabetes Mellitus. Evid. Based Complement. Altern. Med..

[19] Koh J.-H., Kim J.-M., Chang U.-J., Suh H.-J. (2003). Hypocholesterolemic effect of hot-water extract from mycelia of *Cordyceps sinensis*. Biol. Pharm. Bull..

[20] Nagata A., Tajima T., Uchida M. (2006). Supplemental anti-fatigue effects of *Cordyceps sinensis* (tochu-kaso) extract powder during three stepwise exercise of human. Jpn. J. Phys. Fit. Sports Med..

[21] Wang J.M., Shan J.P., Wang S.Q. (2003). Progress of research study on pharmacological action of *Rhodiola rosea*. ACMP Assoc. Chang. Manag. Prof..

[22] Panossian A., Hamm R., Wikman G., Efferth T. (2014). Mechanism of action of Rhodiola, salidroside, tyrosol and triandrin in isolated neuroglial cells: An interactive pathway analysis of the downstream effects using RNA microarray data. Phytomedicine.

[23] Chiang H.M., Chien Y.C., Wu C.H., Kuo Y.H., Wu W.C., Pan Y.Y., Su Y.H., Wen K.C. (2014). Hydroalcoholic extract of *Rhodiola rosea* L. (Crassulaceae) and its hydrolysate inhibit melanogenesis in B16F0 cells by regulating the CREB/MITF/tyrosinase pathway. Food. Chem. Toxicol..

[24] Chiou W.F., Chang P.C., Chou C.J., Chen C.F. (2000). Protein constituent contributes to the hypotensive and vasorelaxant activities of *Cordyceps sinensis*. Life Sci..

[25] Chen C.Y., Hou C.W., Bernard J.R., Chen C.C., Hung T.C., Cheng L.L., Liao Y.H., Kuo C.H. (2014). Rhodiola crenulata- and *Cordyceps sinensis*-Based Supplement Boosts Aerobic Exercise Performance after Short-Term High Altitude Training. High Alt. Med. Biol..

[26] Colson S.N., Wyatt F.B., Johnston D.L., Autrey L.D., Fitzgerald Y.L., Earnest C.P. (2005). *Cordyceps sinensis*- and *Rhodiola rosea*-based supplementation in male cyclists and its effect on muscle tissue oxygen saturation. J. Strength Cond. Res..

[27] Storer T.W., A Davis J., Caiozzo V.J. (1990). Accurate prediction of VO2max in cycle ergometry. Med. Sci. Sports Exerc..

[28] Ahmed M., Henson D.A., Sanderson M.C., Nieman D.C., Zubeldia J.M., Shanely R.A. (2015). *Rhodiola rosea* Exerts Antiviral Activity in Athletes Following a Competitive Marathon Race. Front. Nutr..

[29] Kang D.Z., Hong H.D., Kim K.I., Choi S.Y. (2015). Anti-Fatigue Effects of Fermented *Rhodiola rosea* Extract in Mice. Prev. Nutr. Food Sci..

[30] Ballmann C.G., Maze S.B., Wells A.C., Marshall M.M., Rogers R.R. (2019). Effects of short-term *Rhodiola Rosea* (Golden Root Extract) supplementation on anaerobic exercise performance. J. Sports Sci..

[31] Parisi A., Tranchita E., Duranti G., Ciminelli E., Quaranta F., Ceci R., Cerulli C., Borrione P., Sabatini S. (2010). Effects of chronic Rhodiola Rosea supplementation on sport performance and antioxidant capacity in trained male: Preliminary results. J. Sports Med. Phys. Fit..

[32] Walker T.B., Altobelli S.A., Caprihan A., Robergs R.A. (2007). Failure of *Rhodiola rosea* to alter skeletal muscle phosphate kinetics in trained men. Metabolism.

[33] Jowko E., Sadowski J., Dlugolecka B., Gierczuk D., Opaszowski B., Cieslinski I. (2018). Effects of Rhodiola rosea supplementation on mental performance, physical capacity, and oxidative stress biomarkers in healthy men. J. Sport Health Sci..

[34] Hirsch K.R., Smith-Ryan A.E., Roelofs E.J., Trexler E.T., Mock M.G. (2017). *Cordyceps militaris* improves tolerance to high-intensity exercise after acute and chronic supplementation. J. Diet Suppl..

[35] Chen S., Li Z., Krochmal R., Abrazado M., Kim W., Cooper C.B. (2010). Effect of Cs-4 (*Cordyceps sinensis*) on Exercise Performance in Healthy Older Subjects: A Double-Blind, Placebo-Controlled Trial. J. Altern. Complement. Med..

[36] Yan F., Wang B., Zhang Y. (2014). Polysaccharides from Cordyceps sinensis mycelium ameliorate exhaustive swimming exercise-induced oxidative stress. Pharm. Biol..

[37] Kumar R., Negi P., Singh B., Ilavazhagan G., Bhargava K., Sethy N.K. (2011). Cordyceps sinensis promotes exercise endurance capacity of rats by activating skeletal muscle metabolic regulators. J. Ethnopharmacol..

[38] Sen C.K., Marin E., Kretzschmar M., Hänninen O. (1992). Skeletal muscle and liver glutathione homeostasis in response to training, exercise, and immobilization. J. Appl. Physiol..

[39] Powers S.K., Criswell D., Lawler J., Martin D., Lieu F.K., Ji L.L., Herb R.A. (1993). Rigorous exercise training increases superoxide dismutase activity in ventricular myocardium. Am. J. Physiol. Circ. Physiol..

[40] Evelo C.T.A., Palmen N.G.M., Artur Y., Janssen G.M.E. (1992). Changes in blood glutathione concentrations, and in erythrocyte glutathione reductase and glutathione S-transferase activity after running training and after participation in contests. Graefe’s Arch. Clin. Exp. Ophthalmol..

[41] Covas M.I., Elosua R., Fito M., Alcantara M., Coca L., Marrugat J. (2002). Relationship between physical activity and oxidative stress biomarkers in women. Med. Sci. Sports Exerc..

[42] Bergholm R., Mäkimattila S., Valkonen M., Liu M.-L., Lahdenperä S., Taskinen M.-R., Sovijärvi A., Malmberg P., Yki-Järvinen H. (1999). Intense physical training decreases circulating antioxidants and endothelium-dependent vasodilatation in vivo. Atherosclerosis.

[43] De Sanctis R., De Bellis R., Scesa C., Mancini U., Cucchiarini L., Dachà M. (2004). In vitro protective effect of *Rhodiola rosea* extract against hypochlorous acid-induced oxidative damage in human erythrocytes. BioFactors.

[44] Wing S.L., Askew E.W., Luetkemeier M.J., Ryujin D.T., Kamimori G.H., Grissom C.K. (2003). Lack of effect of Rhodiola or oxygenated water supplementation on hypoxemia and oxidative stress. Wilderness Environ. Med..

[45] Chen T.S., Liou S.Y., Chang Y.L. (2008). Antioxidant Evaluation of Three Adaptogen Extracts. Am. J. Chin. Med..

[46] Després J.P., Pouliot M.C., Moorjani S., Nadeau A., Tremblay A., Lupien P.J., Thériault G., Bouchard C. (1991). Loss of abdominal fat and metabolic response to exercise training in obese women. Am. J. Physiol. Metab..

[47] Leon A.S., Conrad J., Hunninghake D.B., Serfass R. (1979). Effects of a vigorous walking program on body composition, and carbohydrate and lipid metabolism of obese young men. Am. J. Clin. Nutr..

[48] Woo R., Garrow J.S., Pi-Sunyer F.X. (1982). Voluntary food intake during prolonged exercise in obese women. Am. J. Clin. Nutr..

[49] Tremblay A., Simoneau J.A., Bouchard C. (1994). Impact of exercise intensity on body fatness and skeletal muscle metabolism. Metabolism.

[50] Lin K.T., Hsu S.W., Lai F.Y., Chang T.C., Shi L.S., Lee S.Y. (2016). Rhodiola crenulata extract regulates hepatic glycogen and lipid metabolism via activation of the AMPK pathway. BMC Complement. Altern. Med..

[51] Pomari E., Stefanon B., Colitti M. (2015). Effects of Two Different *Rhodiola rosea* Extracts on Primary Human Visceral Adipocytes. Molecules.

[52] Chen X., Wu Y., Yang T., Wei M., Wang Y., Deng X., Shen C., Li W., Zhang H., Xu W. (2016). Salidroside alleviates cachexia symptoms in mouse models of cancer cachexia via activating mTOR signalling. J. Cachexia Sarcopenia Muscle.

[53] Zhong X., Lin R., Li Z., Mao J., Chen L. (2014). Effects of Salidroside on cobalt chloride-induced hypoxia damage and mTOR signaling repression in PC12 cells. Biol. Pharm. Bull..

[54] Song J., Wang Y., Teng M., Cai G., Xu H., Guo H., Liu Y., Wang D., Teng L. (2015). Studies on the Antifatigue Activities of *Cordyceps militaris* Fruit Body Extract in Mouse Model. Evid. Based Complement. Altern. Med..

[55] Urso M.L., Clarkson P.M. (2003). Oxidative stress, exercise, and antioxidant supplementation. Toxicology.

[56] Gomez-Cabrera M.-C., Domenech E., Romagnoli M., Arduini A., Borras C., Pallardo F.V., Sastre J., Viña J. (2008). Oral administration of vitamin C decreases muscle mitochondrial biogenesis and hampers training-induced adaptations in endurance performance. Am. J. Clin. Nutr..

[57] Paulsen G., Cumming K.T., Holden G., Hallén J., Rønnestad B.R., Sveen O., Skaug A., Paur I., Bastani N.E., Østgaard H.N. (2014). Vitamin C and E supplementation hampers cellular adaptation to endurance training in humans: A double-blind, randomised, controlled trial. J. Physiol..

[58] Morrison D., Hughes J., Della Gatta P.A., Mason S., Lamon S., Russell A.P., Wadley G.D. (2015). Vitamin C and E supplementation prevents some of the cellular adaptations to endurance-training in humans. Free Radic. Biol. Med..

[59] Nikolaidis M.G., Kerksick C.M., Lamprecht M., McAnulty S.R. (2012). Does vitamin C and E supplementation impair the favorable adaptations of regular exercise?. Oxid. Med. Cell Longev..

[60] Yuan Y., Wu S.J., Liu X., Zhang L.L. (2013). Antioxidant effect of salidroside and its protective effect against furan-induced hepatocyte damage in mice. Food Funct..

[61] Gudkov S.V., Shtarkman I.N., Smirnova V.S., Chernikov A.V., Bruskov V.I. (2006). Guanosine and inosine display antioxidant activity, protect DNA in vitro from oxidative damage induced by reactive oxygen species, and serve as radioprotectors in mice. Radiat. Res..

[62] Maggirwar S., Dhanraj D., Somani S., Ramkumar V. (1994). Adenosine Acts as an Endogenous Activator of the Cellular Antioxidant Defense System. Biochem. Biophys. Res. Commun..

[63] Ramkumar V., Hallam D.M., Nie Z. (2001). Adenosine, Oxidative Stress and Cytoprotection. Jpn. J. Pharmacol..

[64] Chen J., Zhang W., Lu T., Li J., Zheng Y., Kong L. (2006). Morphological and genetic characterization of a cultivated Cordyceps sinensis fungus and its polysaccharide component possessing antioxidant property in H22 tumor-bearing mice. Life Sci..

